# Assessment of beam‐matched linacs quality/accuracy for interchanging SBRT or SRT patient using VMAT without replanning

**DOI:** 10.1002/acm2.12492

**Published:** 2018-11-07

**Authors:** Zhengzheng Xu, Gregory Warrell, Soyoung Lee, Valdir Colussi, Yiran Zheng, Rodney Ellis, Mitchell Machtay, Tarun Podder

**Affiliations:** ^1^ Department of Radiation Oncology University Hospitals Seidman Cancer Center Cleveland OH USA; ^2^ School of Medicine Case Western Reserve University Cleveland OH USA

**Keywords:** beam‐matching, SBRT, small field dosimetry

## Abstract

**Purpose:**

Dosimetric accuracy is critical when switching a patient treated with stereotactic body radiation therapy (SBRT) or stereotactic fractionated radiotherapy (SRT) among beam‐matched linacs. In this study, the dose delivery accuracy of volumetric modulated arc therapy (VMAT) plans for SBRT/SRT patients were evaluated on three beam‐matched linacs.

**Method:**

Beam data measurements such as percentage depth dose (PDD_10_), beam profiles, output factors, and multi‐leaf collimator (MLC) leaf transmission factor for 6 MV photon beam were performed on three beam‐matched linacs. The Edge™ diode detector was used for measurements of beams of field size less than 5 × 5 cm^2^. Ten lung and 15 brain plans were generated using VMAT with the same beam model. Modulation complexity score of the VMAT plan (MCSv) was used as a plan complexity indicator. Doses were measured using ArcCHECK™ and GafChromic™ EBT3 films. The measurements were compared with calculated doses through absolute dose gamma comparison using 3%/2 mm and 2%/2 mm criteria. Correlation between difference in passing rates among beam‐matched linacs and MCSv was evaluated using the Pearson coefficient. Point doses were measured with the A1SL micro ion chamber.

**Results:**

Difference in beam outputs, beam profiles, and MLC leaf transmission factors of beam‐matched linacs were all within ±1%, except the difference in output factor for 1 × 1 cm^2^ field between linac 1 and 3 (1.3%). For all 25 cases, passing rates of measured doses on three linacs were all higher than 90% when using 2%/2 mm gamma criteria. The average difference in point dose measurements among three beam‐matched linacs was 0.1 ± 0.2% (*P *> 0.05, one‐way ANOVA).

**Conclusion:**

Minimal differences in beam parameters, point doses, and passing rates among three linacs proved the viability of swapping SBRT/SRT using VMAT among beam‐matched linacs. The effect of plan complexity on passing rate difference among beam‐matched linacs is not statistically significant.

## 1 INTRODUCTION

In any high‐volume/high‐throughput clinical center switching patients among available linacs can be very convenient and highly desirable. It may be required due to sudden breakdown of any linac, unexpected high patient load, or other reasons. However, switching a patient treated with stereotactic body radiation therapy (SBRT) or stereotactic fractionated radiotherapy (SRT) to another linac needs very careful considerations. For Varian's “fine beam matching” of photon beams, depth of maximum dose along the central axis is matched within ±1.5 mm and the difference in percentage depth dose at 10 cm depth (PDD_10_) shall be within ±0.5%. For beam profiles, any dose point within the central 80% of the radial and transverse axis, normalized to the central axis at 10 cm depth of water shall be within ±2% of the average of the measured values at that point. The output at the depth of maximum dose in water shall be within ±1% of the average.^1^, ^2^, ^3^ Elekta's factory matching for photon beam requires PDD_10_ to be within ±1% for the group of beam‐matched linacs. Then, for beam profiles of 10 × 10 cm^2^ and 30 × 30 cm^2^ field sizes at 10 cm depth, any averaged point dose (average of the measurements over 1 cm range from that point) within the region covering 80% of full width at half maximum (FWHM) shall be within a 2% difference when compared to the same points from profiles of other beam‐matched linacs.^4^, ^5^ Beam‐matched linacs have almost the same dosimetric characteristics and can be represented as one set of beam parameters in the treatment planning system (TPS). Having beam‐matched linacs can not only increase the flexibility in patient treatment but also reduce the social and economic effects caused by machine down time.^1^

Small fields are often used in lung SBRT and brain SRT for delivering escalated dose to the target while limiting the toxicity of critical structures.^6^ As suggested by Institute of Physics and Engineering in Medicine (IPEM) Report 103 and International Atomic Energy Agency (IAEA) Report 483, field sizes that result in loss of lateral charged particle equilibrium or partial occlusion of the primary photon source by the collimating devices on the beam axis, are under the category of small photon fields.^6^, ^7^, ^8^ For fields smaller than 1.5 cm across, field size changes of 1 mm can lead to central axis dose differences greater than one percent^8^; and for fields 1 cm across, sub‐millimetre changes in field size can lead to dose uncertainties of several percent.^9^, ^10^ Volumetric modulated arc therapy (VMAT) has been increasingly used in lung and brain radiotherapy for its capability of normal tissue dose sparing without sacrificing target dose coverage.^11^, ^12^, ^13^ The multi‐leaf collimator (MLC) is an essential part in VMAT planning for beam modulation. As for VMAT of small fields, the positioning accuracy of MLC leaves plays an important role in shaping the steep dose gradient in order to deliver high dose to the target while maintaining low dose to normal tissues.

Dosimetric accuracy of a VMAT plan is critical when switching the SBRT/SRT patient among beam‐matched linacs. Several studies have reported beam‐matching results and beam data reproducibility for Varian, Elekta, and Siemens linacs.^2^, ^14^, ^15^, ^16^ Gagneur et al. analyzed patient‐specific quality assurance passing rates using 3%/3 mm gamma criteria to verify if three beam‐matched linacs were performing dosimetrically similar when delivering intensity modulated radiation therapy (IMRT) plans.^17^ However, so far we know, no patient‐level dosimetric study on VMAT plans of small fields delivered on beam‐matched linacs is available in published literature. In our study, all selected VMAT plans are of equivalent jaw openings less than 4.0 × 4.0 cm^2^. The dosimetric accuracy of VMAT plans delivered on three beam‐matched linacs was evaluated.

## 2 METHODS

Three Elekta^™^ (Elekta, Stockholm, Sweden) linear accelerators were installed in our institution including one Infinity^™^ (i.e., linac 1) and one Synergy^™^ (i.e., linac 2), both have 6 and 15 MV photon energies and are equipped with Agility^™^ heads (80 MLC leaf pairs of 5 mm leaf width). The third linac is a Versa^™^ (i.e., linac 3) that has 6 and 10 MV photon energies as well as 80 MLC leaf pairs of 5 mm width. All these considered beams are with flattening filter.

The 6 MV Photon beam of linac 3 was selected as the reference for tuning of beams of linac 1 and linac 2. Per institutional acceptance criteria, difference in PDD_10_ among matched linacs should be within ±1%. As for flatness and symmetry of beam profiles, instead of using averaged point dose, any point dose within the 80% of FWHM region shall be within a 2% difference window. During beam‐matching, all beam commissioning measurements were performed for matched photon energies. The commissioning data for 6 MV photon beam of linac 3 were imported in Pinnacle TPS (v9.10, Philips Medical System, MA, USA) as the standard beam model for all three beam‐matched linacs. Although there are no specific limits on the segment monitor unit (MU) and equivalent field size in Pinnacle TPS for VMAT planning, the smallest beam model applied was 2 × 2 cm^2^. Therefore, the smallest target allowed for VMAT treatment should have at least a 2 × 2 cm^2^ equivalent square field size per degree of gantry rotation from the beam's eye view. In addition, the minimum MU per degree was 0.3.

### 2.A Beam profile and output factor measurements

Beam data measurements of percentage depth dose profiles, beam profiles (flatness and symmetry of both crossplane and inplane beam profiles), output factors, and MLC leaf transmission factors were performed on all three beam‐matched linacs. Beam profiles were measured for 2 × 2 cm^2^, 5 × 5 cm^2^, 10 × 10 cm^2^, and 30 × 30 cm^2^ field sizes at 1.5 and 10 cm depths. PDD_10_ were measured for beams of 2 × 2 cm^2^, 5 × 5 cm^2^, 10 × 10 cm^2^, and 30 × 30 cm^2^ field sizes. Depth dose and beam profile measurements were conducted using the IBA Blue Phantom^™^ scanning phantom system (IBA dosimetry, Germany). For photon beams used in SBRT, American Association of Physicists in Medicine (AAPM) Task Group 101 recommend that the detector should have a spatial resolution of higher than 1 mm for basic dosimetry data measurement.^18^ The IBA CC13 ion chamber of 0.13 cm^3^ cavity volume was used for measurements including beam profiles and output factors of field sizes larger than or equal to 5 × 5 cm^2^; while the Edge^™^ diode detector (Sun Nuclear Corporation, FL, USA) of 0.019 mm^3^ volume was used for beams of field sizes less than 5 × 5 cm^2^ (e.g., 2 × 2 cm^2^ and 5 × 5 cm^2^). The relative output factor was measured at 10 cm depth and 100 cm source to surface distance for field sizes ranging from 1 × 1 cm^2^ to 30 × 30 cm^2^. To minimize the energy variation on output factor measurements, a 3 × 3 cm^2^ was selected as an intermediate or reference field size to normalize the diode against the CC13 ion chamber measurements following the “Daisy‐chain” approach in eq. (1).

(1) $$OF_{\mathrm{edge}}=\frac{M_{edge_{-}FS}}{M_{edge_{-}3\times 3}}\times \frac{M_{CC13_{-}3\times 3}}{M_{CC13_{-}10\times 10}}$$

Measurements for output factor of specific field size were repeated three times with diode and ion chamber to evaluate the consistency of MU delivery and detector measurements.

MLC leaf transmission (average of the intraleaf and interleaf leakage) was measured using PTW (Feiburg, Germany) N31003 farmer chamber placed at 100 cm source to axis distance and 1.5 cm depth in the solid water phantom with the chamber long axis parallel to the MLC leaf motion direction. The MLC leaf transmission factor was the ratio of the exposure measured for an open field with 10 × 10 cm^2^ jaw size and the exposure measured with the same jaw size but MLC leaves closed. Beam data comparison was evaluated by calculating the difference between the measurement data of two linacs (linac 1 and 2) to the reference linac (linac 3).

### 2.B Dose measurements for clinical SBRT and SRT plans

**Figure 1:**
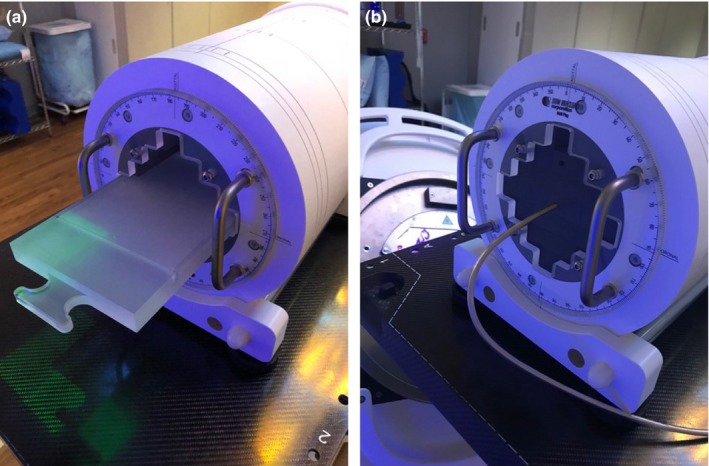
Illustration of ArcCHECK with (a) film holder and (b) A1SL micro ion chamber inserted for planar and point dose measurements.

Ten lung cases were prescribed with 50 Gy in five fractions and 15 brain cases including primary and metastatic tumors were prescribed with 30 Gy or 25 Gy in five fractions. All the VMAT plans were generated in Pinnacle TPS using the same 6 MV beam model. Lung VMAT plans used two full arcs with jaw sizes ranging from 2.4 × 3.1 cm^2^ to 4.3 × 3.9 cm^2^. Based on the location of brain tumor, VMAT plans used either multiple non‐coplanar arcs (e.g., one full arc and one vertex with couch kick) or multiple coplanar partial arcs (e.g., two to four partial arcs for posteriorly located targets), with jaw sizes ranging from 1.6 × 3.0 cm^2^ to 3.7 × 4.6 cm^2^. All the VMAT plans were measured using ArcCHECK^™^ cylindrical diode array system (Sun Nuclear, FL, USA) and Gafchromic™ EBT3 films (Ashland Inc., NJ, USA). The films were placed in the acrylic film holder specifically designed for ArcCHECK [Fig. 1(a)]. Films used for VMAT planar dose measurements and absolute dose calibration were from the same lot and scanned in the same portrait orientation with 300 dpi (dots per inch) resolution. ArcCHECK and film measurements were compared with the TPS calculated planar doses through absolute dose gamma comparison using 3%/2 mm and 2%/2 mm criteria. Point doses were measured and used as another independent verification of absolute dose. The Extradin A1SL (Standard Imaging, Inc., WI, USA) micro ion chamber of 0.053 cm^3^ volume was placed in the middle of the acrylic insert inside the ArcCHECK [Fig. 1(b)]. Differences in gamma passing rates of ArcCHECK measurements and point dose measurements among three linacs were analyzed using one‐way ANOVA. ArcCHECK and ion chamber measurements were repeated on three different days at all three linacs to evaluate the measurement uncertainty. A *P*‐value less than 0.05 was considered statistically significant.

The modulation complexity score of VMAT (MCSv) was applied to evaluate the plan complexity degree of a VMAT plan.^19^, ^20^ MCSv ranges from 0 to 1, and it approaches 0 for increasing degree of VMAT plan modulation. The correlation between difference in passing rates of each plan among matched linacs and its modulation complexity was evaluated using Pearson correlation coefficient.

## 3 RESULTS

The variation in repeated output factor measurements using diode and ion chamber for three beam‐matched linacs were all within ±0.4% of the average. Differences in beam output factors of 2 × 2 cm^2^ to 30 × 30 cm^2^ field sizes among three beam‐matched linacs were all less than 1% (Table 1). The maximum difference in output factor was 1.3% which was the difference in output factor of 1 × 1 cm^2^ field size between linac 1 and 3. Differences in PDD_10_ and MLC leaf transmission factors among three linacs were all less than 0.6% (Table 2). Beam profiles measurements, including flatness and symmetry, of different field sizes at 1.5 and 10 cm depths for beam‐matched linacs were listed in Table 3. The average difference in flatness and symmetry among three linacs were 0.3 ± 0.8% and 0.1 ± 0.3%, respectively, for beam profiles at 1.5 cm depth; while 0.1 ± 0.7% and 0.2 ± 0.3%, respectively, for beam profiles at 10 cm depth. The maximum difference was 1.8% which was the inplane flatness for 2 × 2 cm^2^ field between linac 1 and linac 3 at 1.5 cm depth.

**Table 1:** Output factors for 6 MV photon beam of different field sizes for three beam‐matched linacs

Field size (cm × cm)	Linac 1	Linac 2	Linac 3	Maximal difference (%)
1 × 1	0.699	0.705	0.708	1.3
2 × 2	0.808	0.806	0.810	0.5
3 × 3	0.847	0.845	0.844	0.4
4 × 4	0.876	0.876	0.875	0.1
5 × 5	0.905	0.907	0.906	0.2
10 × 10	1.000	1.000	1.000	0.0
20 × 20	1.093	1.095	1.094	0.1
30 × 30	1.136	1.137	1.137	0.1

**Table 2:** Difference in PDD_10_ for beams and MLC leaf transmission factors among beam‐matched linacs

6 MV	Linac 1	Linac 2	Linac 3	Maximal difference
PDD_10_ (2 × 2 cm^2^)	60.5%	60.6%	60.1%	0.5%
PDD_10_ (5 × 5 cm^2^)	63.9%	63.3%	63.9%	0.6%
PDD_10_ (10 × 10 cm^2^)	67.3%	67.3%	67.4%	0.2%
PDD_10_ (30 × 30 cm^2^)	71.2%	71.5%	71.6%	0.4%
MLC leaf transmission	0.7%	0.4%	0.6%	0.3%

**Table 3:** Beam flatness and symmetry of different field sizes and depths for beam‐matched linacs

	Linac 1				Linac 2				Linac 3			
	Crossplane		Inplane		Crossplane		Inplane		Crossplane		Inplane	
d: 1.5 cm	Flatness (%)	Symmetry (%)	Flatness (%)	Symmetry (%)	Flatness (%)	Symmetry (%)	Flatness (%)	Symmetry (%)	Flatness (%)	Symmetry (%)	Flatness (%)	Symmetry (%)
2 × 2	6.8	0.6	9.5	0.7	6.2	0.6	11.0	0.3	5.2	0.5	11.3	0.4
5 × 5	2	0.2	2	0.2	2.5	0.0	1.8	0.5	1.2	0.6	2.1	0.1
10 × 10	0.4	0.3	1.4	0.1	1.8	0.4	1.8	0.6	0.7	0.3	0.8	0.1
30 × 30	3	0.2	3.9	0.5	2.0	0.1	3.0	0.1	2.5	0.1	2.9	0.1
d: 10 cm												
2 × 2	7.2	0.4	10.3	0.7	6.5	0.4	11.6	0.6	5.8	0.6	11.9	0.0
5 × 5	2.8	0.6	3.2	0	2.8	0.8	3.7	0.5	2.2	0.4	3.3	0.1
10 × 10	2.1	0.8	2.3	0.6	1.9	0.0	2.6	0.1	2.3	0.2	2.0	0.0
30 × 30	1.8	0.5	1.4	0.2	1.7	0.1	1.6	0.2	1.4	0.0	1.8	0.2

d, depth.

**Figure 2:**
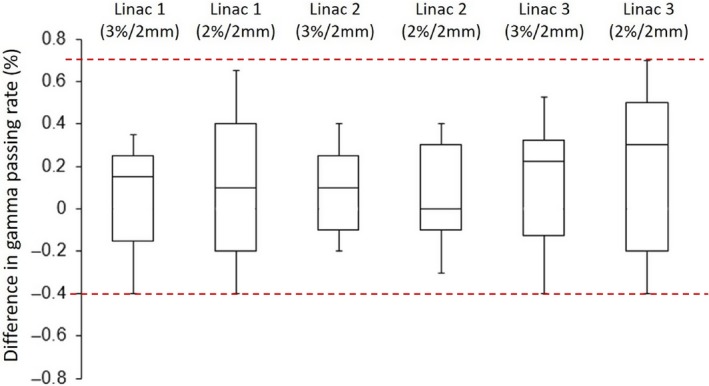
Distributions of gamma passing rate variation of SBRT/SRT VMAT plans delivered on three beam‐matched linacs and measured with ArcCHECK on three different days.

**Figure 3:**
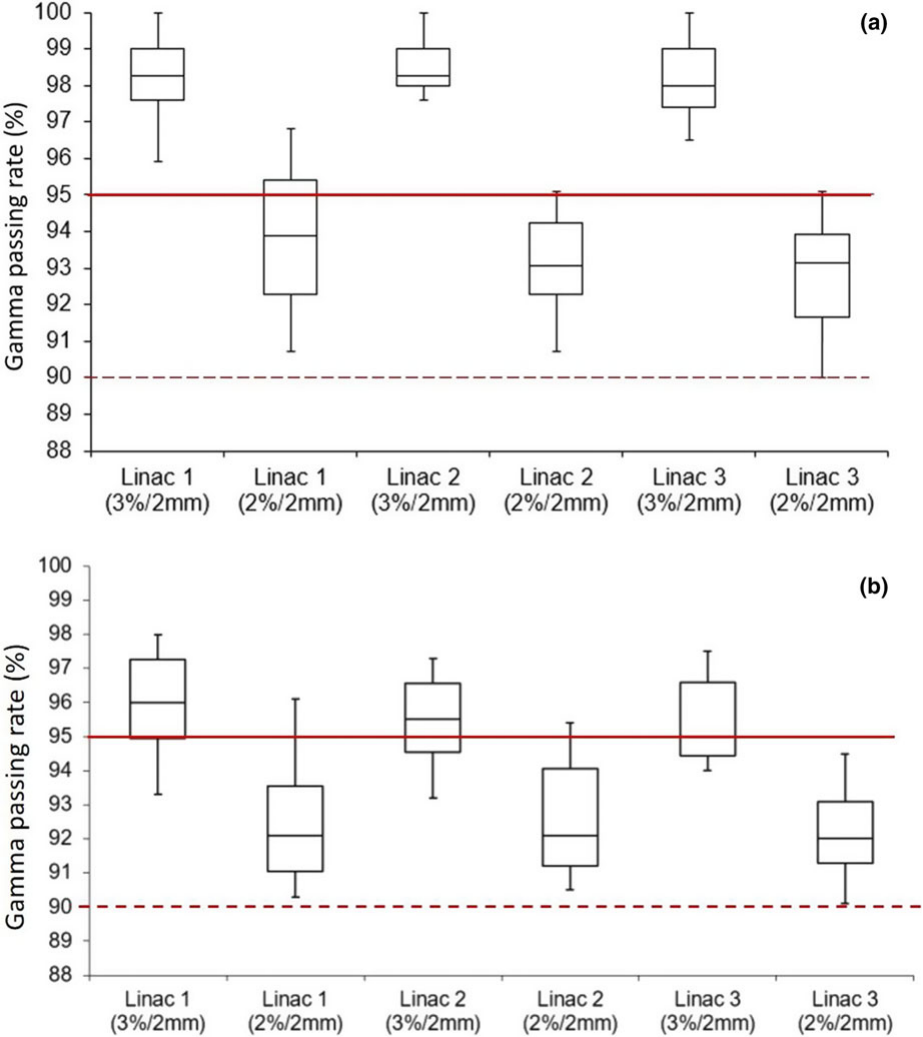
Distributions of absolute dose gamma passing rates of SBRT/SRT VMAT plans delivered on three beam‐matched linacs. (a) Passing rates of ArcCHECK measurements; (b) Passing rates of EBT3 film measurements. Solid red line: 95% passing rate threshold for 3%/2 mm gamma criteria. Dashed red line: 90% passing rate threshold for 2%/2 mm gamma criteria.

For all 25 cases, variation in passing rates of repeated ArcCHECK measurements acquired from three linacs on three different days were demonstrated in Fig. 2. Passing rates variations of each plan delivered on three linacs were all within the ranges of ±0.4% to ±0.7% when applied 3%/2 and 2%/2 mm gamma criteria, respectively. Passing rates of ArcCHECK measurements on three linacs were all higher than 95% and 90% using 3%/2 and 2%/2 mm gamma criteria, respectively [Fig. 3(a)]; while passing rates of film measurements were all higher than 90% using 3%/2 and 2%/2 mm gamma criteria [Fig. 3(b)]. There was small difference in passing rates of ArcCHECK measurements among three linacs while either using 3%/2 mm criteria (*P* > 0.05, one‐way ANOVA) or 2%/2 mm criteria (*P* > 0.05, one‐way ANOVA). Linac 1 and 3 demonstrated the maximum difference in passing rates of ArcCHECK measurements and ion chamber measurements among the group (Table 4). As demonstrated in Table 4, the average difference in absolute point doses between ion chamber measurements and TPS calculations was −1.5 ± 0.8% indicating lower measurements compared to TPS calculations. The average difference in point dose measurements among three matched linacs was 0.1 ± 0.5% (*P* > 0.05, one‐way ANOVA). However, none of these differences was statistically significant.

**Table 4:** Difference in passing rates of ArcCHECK and film measurements and point doses among beam‐matched linacs

	L1 vs L2	L1 vs L3	L2 vs L3	Mean ± SD
3%/2 mm (ArcCHECK)	0.2 ± 0.5%	0.3 ± 0.6%	0.2 ± 0.3%	0.3 ± 0.5%
r^+^	0.15	0.23	0.19	–
2%/2 mm (ArcCHECK)	0.6 ± 1.8%	0.9 ± 2.1%	0.8 ± 1.5%	0.8 ± 1.7%
r	0.19	0.20	0.24	–
3%/2 mm (Film)	1.2 ± 1.8%	1.7 ± 1.5%	1.5 ± 1.2%	1.6 ± 1.3%
r	0.23	0.14	0.26	–
2%/2 mm (Film)	2.3 ± 1.6%	1.9 ± 2.2%	2.5 ± 1.7%	2.2 ± 1.9%
r	0.20	0.16	0.21	–
Point dose difference 1^a^	−1.3 ± 0.8%	−2.1 ± 0.6%	−0.9 ± 1.1%	−1.5 ± 0.8%
r	0.22	0.17	0.15	–
Point dose difference 2^b^	−0.1 ± 0.5%	0.2 ± 0.3%	0.1 ± 0.3%	0.1 ± 0.5%
r	0.14	0.21	0.11	–

L1, linac 1; L2, linac 2; L3, linac 3; (+) r value, Pearson correlation coefficient.

a Point dose difference 1 is the difference between measured dose with ion chamber and TPS calculation.

b Point dose difference 2 is the difference in measured doses between two beam‐matched linacs.

MCSv of all VMAT plans ranged from 0.34 to 0.57. The correlation coefficients for the difference in passing rates of ArcCHECK, film and ion chamber measurements among three linacs and MCSv were 0.15–0.24, 0.14–0.26, and 0.11–0.22, respectively (Table 4).

## 4 DISCUSSION

The average difference in measurements of beam profile, output factor (except for 1 × 1 cm^2^), and MLC leaf transmission factor among three beam‐matched linacs were less than ±1% indicating that these linacs are clinically identical. The difference in output factor for 1 × 1 cm^2^ field size beam between linac 1 and 2 was 1.3%. However, model for beam of 1 × 1 cm^2^ field size was not applied for VMAT planning in Pinnacle TPS and the smallest target allowed in our institution for VMAT treatment should have at least a 2 × 2 cm^2^ equivalent square field size for each control point.

Beam of 1 × 1 cm^2^ field size for 6 MV is considered as very small field size.^8^ Even with stereotactic detectors, careful detector‐phantom setup and detailed dose corrections, one might still find more than 10% discrepancies among the measurements of very small fields (<1 cm in diameter).^18^ The major perturbations were caused by the volume averaging effect and the difference between the mass density of the detector and that of the medium.^9^ Studies have reported that the effect of volume averaging for detectors up to 3 mm in size was only noticeable at field sizes of less than 8 mm.^9^, ^21^ The Edge detector can minimize the volume averaging effect for output measurements of beams with very small field sizes. In addition, the detector has a comparatively high sensitivity, and results in lower signal noise or standard deviation of signal. Then, the output of field sizes around 1 × 1 cm^2^ is affected by jaw positioning accuracy. Charles et al.^9^ stated that the error in output factor caused by a 1 mm field size error at a field size of 1.2 × 1.2 cm^2^ was 1.7%, and increased sharply with decreasing field size below 1.2 × 1.2 cm^2^. Kairn et al.^14^ evaluated the variation of small field collimation for eight beam‐matched Varian linacs. They reported that, for fields collimated by MLCs, the field sizes were consistent among beam‐matched linacs while variation from the nominal values were higher compared to fields defined by orthogonal jaws. For fields collimated by MLCs, the apertures were approximately 0.3 mm narrower than the nominal field size for the fields less than 1 × 1 cm^2^ and these field size differences had substantial effects on measured output factor.

The volume of each diode in the ArcCHECK is 0.019 mm^3^ (0.8 × 0.8 mm^2^, N‐type), which is of the same volume as the Edge diode detector (0.019 mm^3^, 0.8 × 0.8 mm^2^, N‐type). Therefore, sensitivity and volume averaging effect of both detectors are assumed to be very close. The difference in absolute dose calibration for three linac for 10 × 10 cm^2^ field at dmax for 6 MV beams was within 0.1% (0.999–1.000 cGy/MU). Difference in output factors of field sizes ranging from 2 × 2 cm^2^ to 10 × 10 cm^2^ among three linacs were all less than 0.5%. Underwood et al. stated that although detectors either under‐respond or over‐respond on output factors, in small fields, this is actually compensated by an opposing over‐response or under‐response, respectively, in the profile tails.^22^ As a result, by using the same detector, integral dose measurement of a VMAT plan of small field might not need additional output correction factors.^8^, ^22^ Gersh et al. reported that, for lung SBRT using VMAT plans, mean doses to the target and normal structures using beam models measured with CC13 ion chamber and Edge detector were all within 1%.^23^

In this study, all passing rates of film measurements were higher than 90% indicating accurate beam modeling and dose delivery. Average passing rates of film measurements were lower than those of ArcCHECK measurements mainly due to uncertainty in software‐based image registration between scanned films and TPS calculations for gamma analysis. Small differences in repeated ArcCHECK and point dose measurements among three beam‐matched linacs demonstrated consistent phantom setup and accurate dose delivery. Measured dose using A1SL ion chamber was lower than TPS calculations due to the volume averaging effect. Compared with the other two linacs (linac 1 and 3), doses delivered by linac 2 were closer to TPS calculations in terms of higher average passing rates and lower standard deviation in passing rates (ArcCHECK measurements with 3%/2 mm criteria). Therefore, it could be used as the first choice for a backup linac.

During clinical treatment, if the patient receiving SBRT needs to be switched from the primary treating linac to a backup beam‐matched linac, treatment workflow for backup linac is the same as the one for the first fraction of SBRT treatment. The couch index from the primary linac will be used for initial setup at the backup linac along with laser system assistance. The cone beam computed tomography (CBCT) scan is then performed and compared to the planning CT to best match the treating area. After image registration, translational, and rotational shifts are applied and compensated by HexaPOD™ treatment couch (Elekta, Stockholm, Sweden). One limitation of this phantom study is that the setup was based matching the markers on ArcCHECK with the wall and ceiling lasers without further CBCT assistance. Measurements are assumed to be consistent among three linacs based on laser alignment since there is no internal motion or anatomical changes in the phantom. During gamma analysis, because no auto‐alignment was applied, small differences in passing rates among measurements from three linacs demonstrate consistent setup with lasers. However, in clinical treatment, patient motion, and image registration variation with CBCT will affect the dose delivery accuracy when switching to a backup linac. Further study will be conducted on the dosimetric evaluation for patients treated on the backup linacs with CBCT alignment. Another limitation of this study is that plans using 6 MV flatting filter free (FFF) beam are not included since only one linac (linac 3) has this beam mode.

Weak correlation between MCSv and passing rate variation among beam‐matched linacs indicates that the VMAT modulation complexity has little effect on variation in planar dose measurements acquired from three beam‐matched linacs as long as all beam parameters are within machine tolerance.

## 5 CONCLUSION

The beam‐matched linacs demonstrated good agreement between measurements and TPS calculations for SBRT/SRT VMAT plans with small field/segment sizes. Linac 2 demonstrated marginally better VMAT dose delivery performance in terms of gamma passing rate. The effect of plan complexity on pass rate difference is insignificant. Small differences in beam parameters, point doses and passing rates of measurements among three linacs proved the availability of swapping SBRT/SRT VMAT patients among beam‐matched linacs. This can definitely improve the clinical workflow and maintain high patient throughputs.

## CONFLICT OF INTEREST

No conflicts of interest.
